# Unveiling the Preparation and Characterization of Lercanidipine Hydrochloride in an Oral Solid Self-Nanoemulsion for Enhancing Oral Delivery

**DOI:** 10.7759/cureus.64468

**Published:** 2024-07-13

**Authors:** Haneen M Abdul Hussein, Mowafaq M Ghareeb

**Affiliations:** 1 Department of Pharmaceutics, Ministry of Health and Environment, Babylon Health Directorate, Babylon, IRQ; 2 Department of Pharmaceutics, College of Pharmacy, University of Baghdad, Baghdad, IRQ

**Keywords:** dispersion rate, solid self-nano emulsifying, nephroprotective, bioavailability, lercanidipine hcl

## Abstract

Introduction: Chronic kidney disease (CKD) is becoming increasingly prevalent worldwide, particularly among the elderly, along with an increase in the incidence of hypertension and cardiovascular disorders. Developing lipid-based oral dosage forms with a higher expected bioavailability of antihypertensive drugs with nephroprotective effects poses a challenge. Lercanidipine hydrochloride (LRCH) is a newer type of third-generation dihydropyridine calcium channel blocker that functions as an antihypertensive and has significant nephroprotective effects. Due to its extensive first-pass metabolism, its bioavailability is about 10% and increases to 3-4 times when taken with a high-fat meal. Targeting this drug to the lymphatic system using the solid self-nano-emulsifying drug delivery system (SSNEDDS) is a promising approach for improving LRCH's bioavailability and dispersion rate. SSNEDDS combines the benefits of both liquid self-emulsifying and solid dosage forms, improving drug stability and extending storage time.

Materials and methods: In this study, liquid SNEDDS composed of 10% peppermint oil, 67% Tween 20, and 22.5% propylene glycol was solidified using two adsorbent agent mixtures (SSNEDDS1: Avicel PH 101 and Aerosil 200) and (SSNEDDS2: Avicel PH 102 and Aerosil 200) separately. The prepared formulations were evaluated for powder flow, drug content, and an in-vitro dispersion test in comparison to the brand-marketed tablet as a standard or pure drug. DSC and X-ray diffraction analysis were also used.

Results: The SSNEDDS2 shows excellent flowability, a higher drug content (99.761%), and a significantly higher and faster dispersion rate of 100% within 10 minutes compared to 92% of the marketed LRCH tablet and 18.1% of the pure drug for 60 minutes. The solid-state characterization of the formulation composed of SSNEDDS2 confirmed that the LRCH was in an amorphous form inside the solidified nano system without interacting with the excipient.

Conclusion: This study successfully prepared LRCH using the promising strategy of SSNEDDS as a hard gelatin capsule with a higher dispersion rate. It improved its stability and expected bioavailability compared to the brand-marketed tablet as the standard.

## Introduction

Lercanidipine hydrochloride (LRCH) is 5-O-[1-[3,3-diphenylpropyl(methyl)amino]-2-methylpropan-2-yl] 3-O-methyl 2,6-dimethyl-4-(3-nitrophenyl)-1,4-dihydropyridine-3,5-dicarboxylate; hydrochloride. It has the molecular formula C_36_H_41_N_3_O_6_·HCl [1].

Lercanidipine competitively binds to the dihydropyridine (DHP) site of L-type calcium channels in cardiac and vascular smooth muscle cells, inhibiting the transmembrane influx of calcium ions and producing muscle relaxation, because it is lipophilic, the medicine is rapidly eliminated from the bloodstream and accumulates in the phospholipid bilayer of every cell membrane. Because of this, the medication can reach its target, the L-type calcium channel, which is situated at the DHP location within the double layer of the cell membrane. As a result of that, the drug's effects take a long time to settle in and start working. Hence, the antihypertensive action is gentle and progressive, allowing for great pharmacologic control, and reflex tachycardia is practically nonexistent [2]. This drug has been used for therapeutic purposes across the globe for a long time. Lercanidipine has been introduced in 26 countries worldwide since it was first made available in the Netherlands in 1997. It primarily treats mild to moderate hypertension with a nephroprotective property [3] by directly dilating the efferent and afferent glomerular arteries while keeping the intraglomerular capillary pressure constant [4]. Chronic kidney disease (CKD) can have various causes, but it is characterized by a decrease in renal function, which results in a glomerular filtration rate (GFR) below 60 mL/min/per 1.73 m^2^, or signs of renal injury that last for more than three months. CKD has a high morbidity rate, which can reach 11% to 13% worldwide, making it a major public health problem, especially in high-income economies [5]. It is commonly known that the kidney is a primary organ targeted by hypertension [6]. Lercanidipine has several benefits for patients with CKD. It helps increase the bioavailability of endothelial nitric oxide (NO), which exhibits anti-atherogenic properties. Additionally, it has antioxidant and anti-inflammatory effects, and it also protects the kidneys from angiotensin II-induced damage. Therefore, given these and other benefits, lercanidipine appears to be an ideal choice for CKD patients [7]. Developing safer antihypertensive drugs with nephroprotective effects poses a challenge because of the lack of safe oral medications available for this purpose with high bioavailability. Targeting this drug to the lymphatic system using the solid self-nano-emulsifying drug delivery system (SSNEDDS) is a promising approach for improving LRCH's bioavailability and dispersion rate. SSNEDDS combines the benefits of both liquid self-emulsifying and solid dosage forms, improving drug, stability and extending storage time as well as solid dosage form characteristics such as compact size, portability, ease of handling, and patient compliance [8]. When a drug compound enters the digestive fluids during droplet transit, it disintegrates and is released from the self-nano-emulsifying drug delivery system (SNEDDS). Two primary factors determine the effective release of the medicinal ingredient from SNEDDS: tiny droplet size and polarity of the resultant oil droplets [9]. Adsorption on solid carriers allows liquid SE formulations to become free-flowing powders. The liquid formulation is blended with inert carriers in a blender as part of the simple adsorption procedure. The final powder can be put directly into capsules. There is no change in the drug release property when liquid SNEDDS are solidified into dosage form. Upon expulsion in the gastrointestinal tract, the contents undergo self-emulsification [10]. Recently, there has been a surge of research into the benefits of solid dosage forms, particularly SSNEDDSs, due to their potential advantages.

## Materials and methods

Lercanidipine HCl, Aerosil 200 and Avicel 102 (HyperChem, China), Tween 20 (Chemical Point, Germany), propylene glycol (Barcelona, Spain), peppermint oil, methanol and Avicel PH101 Microcrystalline cellulose pH 101 (Thomas Baker, India) and hydrochloric acid (ReAgent Chemicals, UK). Zandip^®^10 mg film-coated tablet (Recordati, Italy).

Preparation of LRCH-SNEDDS

Lercanidipine HCl is loaded as a self-nano-emulsifying liquid formula before solidification. Based on part 1 of this study, 0.3 g of the selected formula was prepared using the following constituents: 10%, 67%, and 22.5% w/w of peppermint oil, Tween 20, and propylene glycol, respectively, in a 1:3 Smix ratio were combined in a vortex mixer to prepare the blank formulation. The mixture was stirred in a screw-capped glass tube for approximately three minutes to ensure homogeneity and good miscibility before adding the drug. Then, 10 mg LRCH was dissolved in a SNEDDS mixture. The mixture was vortexed for five minutes to ensure proper dissemination and dispersion of the drug. Finally, it was sonicated for 10 minutes to produce a yellow and clear liquid [11].

Solidification preparation of solid LRCH-SNEDDS powder

The LRCH-SSNEDDS powders listed in Table 1 were made by combining 0.3 g of each liquid (LRCH-SNEDDS) with two types of adsorbent combinations in a constant 1:1.25 ratio. The adsorbent mixture (carrier: coating) agents consisted of (Avicel 101 + Aerosil 200) and (Avicel 102 + Aerosil 200). First, the carrier is put in the porcelain mortar, and the LRCH-SNEDDS liquid is slowly dropped on it. Mix them gently with a pestle in one direction for 10 minutes at room temperature. Then, after saturation, we put the coating agent with continued mixing until uniform powder with free flowability was obtained [12]. The formed powder was sieved in a sieve of 250 and left for 48 hours at room temperature then filled in a hard gelatin capsule size 0.

**Table 1:** Components of prepared solid self-nanoemulsion formulations SSNEDDS: solid self-nano-emulsifying drug delivery system

Formula code	Liquid SNEDDS wt. (mg)	Carrier: LSNEDDS ratio	Carrier: coating ratio	Avecil PH 101 (mg)	Avecil PH 102 (mg)	Aerosil 200 (mg)	Total formula wt. (mg)
SSNEDDS1	300	1.25:1	10:1	375	---	37.5	712.5
SSNEDDS2	300	1.25:1	10:1	---	375	37.5	712.5

Characterization of LRCH solid self-nanoemulsion

Powder Flow Properties

Angle of repose (AOR): The repose angle indicates the degree of cohesiveness between particles and was commonly used to determine flow characteristics. This angle was measured using the fixed funnel technique, where the funnel height (h) was adjusted to 2 cm above a horizontal plane. The powder mixtures were then carefully poured onto the surface and freely flowed through the funnel's opening. The end of the funnel would be directly at the peak of the conical pile [13]. To calculate the AOR (θ), the conventional funnel technique was utilized. Pouring a constant amount (3 g) of each preparation through the cone, the funnel height over the bench surface was held at 2 cm. Using the following equation, where h is the height and r is the radius, we were able to determine the AOR after a cone-like pile had formed [14] and interpretation of results should follow Table 2.

**Table 2:** Flow properties according to angle of repose value (USP) Reference: [15]

The angle of repose (degree)	Flow properties according to USP
(25-30)	Excellent
(31-35)	Good
(36-40)	Fair no flow aid needed
(41-45)	Passable (flow aid might be needed, may hang up)
(46-50)	Poor (needs agitation or vibration to improve)
(50-56)	Very poor
More than 66	Very very poor

\begin{equation*} \tan \theta = \frac{h}{r} \end{equation*}

where θ represents the AOR, h represents pile height, and r represents base pile radius.

Bulk and tapped density: Three grams of SSNEDDS powder were put in a 10 ml graduated cylinder. The initial volume of the powder in the cylinder was determined. The cylinder was dropped from a height of 2.5 cm every two seconds onto a hard surface. The tapping continued 100 times until the volume no longer changed. The volume was then measured to determine the tapped density [16], the index of flow properties according to Carr is listed in Table 3.

**Table 3:** Powder flow properties based on Carr's (compressibility) index and Hausner ratio Reference: [17]

Compressibility index	Hausner’s ratio	Flow type
(1-10)	1.00-1.11	Excellent
(11-16)	1.12-1.18	Good
(16- 20)	1.19-1.25	Fair
(21 -25)	1.26-1.34	Passable
(26 -31)	1.35-1.45	Poor
(32-37)	1.46-1.59	Very poor
More than 38	More than 1.60	Very very poor

Bulk and tapped densities were calculated by the following equations.

\begin{equation*} \text{Bulk Density (BD)} = \frac{\text{Blended Powder Weight}}{\text{Volume of Poured Blended Powder}} \end{equation*}

\begin{equation*} \text{Tapped Density (TD)} = \frac{\text{Weight of Powder Blend}}{\text{Tapped Volume of Powder Blend}} \end{equation*}

The compressibility index (Carr’s index) and Hausner’s ratio measurement were calculated by the following equations.

\begin{equation*} \text{Carr's Index} = \left( \frac{\text{TD} - \text{BD}}{\text{TD}} \right) \times 100 \end{equation*}

\begin{equation*} \text{Hausner Ratio} = \frac{\text{TD}}{\text{BD}} \end{equation*}

Drug content determination

Each single hard gelatin capsule of solid LRCH-SNEDDS powder, which is equivalent to 10 mg LRCH, was dispersed in 50 ml of methanol in a conical flask. The mixture was shaken and sonicated continuously for 15 minutes, followed by 15 minutes of centrifugation at 3500 rpm. Then, the supernatant liquid was separated and filtered using a 0.45-micron filter [18]. After that, it was diluted with methanol and tested for drug content by UV spectrophotometry at a wavelength of 237 nm using constructed calibration of serial dilutions of stock solution of 10 mg of lercanidipine HCl in separate solutions of 100 ml methanol or 0.1 N HCl (pH 1.2) containing 0.5% w/v Brij35.

In-vitro dissolution of LRCH from solid self-nano-emulsifying capsules

The in-vitro dispersion profiles of LRCH from solid self-nano-emulsifying capsule formulations were investigated using the USP Dissolution Apparatus-II (paddle type) to assess the impact of different adsorbent mixtures on dispersion rate. A 500 ml of 0.1 N (pH 1.2) hydrochloric acid was used as dispersion media with 0.5% Brij35 to maintain sink conditions at 37 ± 0.5ºC [19]. One capsule equivalent to 10 mg LRCH was placed in the basket, which was rotated at 100 rpm [20]. About 5 ml samples were withdrawn at 2, 4, 6, 8, 10, 20, 30, 40, 50, and 60-minute intervals. Fresh media was added to maintain constant sink conditions, and the withdrawn samples were filtered through a 0.45 μm filter membrane. The amount of drug dissolved was quantified at its maximum absorption wavelength (238 nm) using a UV Spectrophotometer. The dispersion of the brand-marketed tablet that was found in the market and pure drug was also done. For comparison among their dispersion profiles, similarity factor (f2) was used in the DD solver software program.

Additional evaluations of the optimal LRCH solid self-nanoemulsion

The formula that shows the best AOR, powder flowability, drug content, and faster dispersion rate was selected for further advanced analytical characterization, these include powder X-ray diffraction, differential scanning calorimetry (DSC), and morphological evaluation.

DSC

An automatic thermal analysis device (Setaram, DSC-131 evo, France) was used to investigate the thermal properties of pure LRCH, LRCH-SNEDDS, and its physical mixture (a 1:1:1 physical mixture of the specified formula, Avecil PH 102, and Aerosil 200). Each sample was heated from 30°C to 300°C in a non-hermetically sealed pan at 10°C per minute. The analysis was conducted under atmospheric flow conditions [21,22].

Powder X-ray Diffraction

Powder X-ray diffraction (the DX2700BH from China) was utilized to examine the crystalline structure of pure LRCH and LRCH-SNEDDS solid formula. The target elements were metal Cu, filter Kα, 30 m, and A40 kV. The scan was performed over a 2θ range of 5-80° with a wavelength of 1.5406 Å [23].

Stability Study

The study was conducted to evaluate the stability of the LRCH-SSNEDDS hard gelatin capsules and LRCH-SNEDDS oral ampules. The capsule was subjected to storage at three distinct temperatures such as 25°C, 40°C, and 50ºC for three months. The samples were collected periodically in months 1, 2, and 3, and were examined for their physical characteristics and drug concentration [24].

Statistical analysis

The research results were displayed using a three-sample average with standard deviation. To determine if the changes in the applied parameters were significant (P < 0.05) or non-significant (P > 0.05), the data was analyzed using a one-way analysis of variance (ANOVA) with a significance level of P < 0.05.

## Results

Liquid formulation for the solidification process

**Figure 1:**
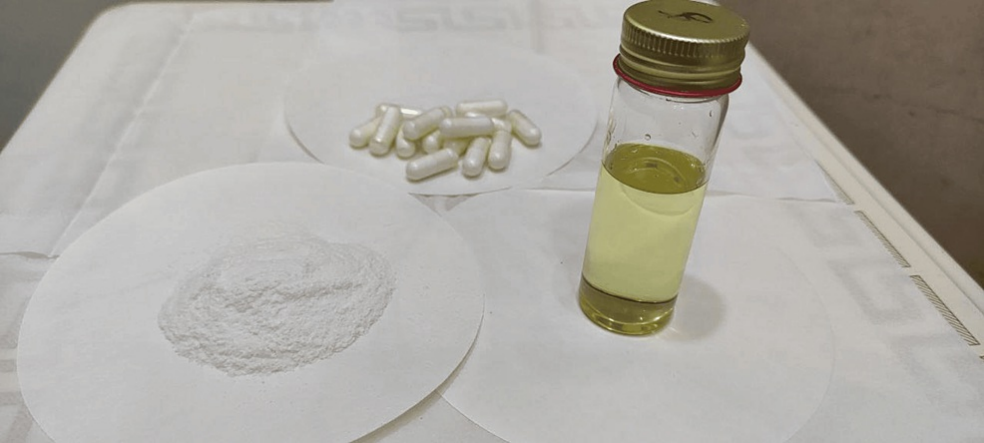
Solidification steps from liquid SSNEDDS formulation SSNEDDS: solid self-nano-emulsifying drug delivery system

Based on part 1 of the study, the solubility of LRCH in various nanoemulsion media was analyzed; peppermint oil, Tween 20, and propylene glycol were chosen as oils, surfactants, and co-surfactants, respectively, according to their maximum dissolving capacity and their excellent miscibility and stability with each other [25]‏. The selected nanoemulsion formula showed a higher dispersion rate with higher drug content and smaller globule size upon dispersion with water, providing O/W nanoemulsion within a shorter emulsification time. We used the simple solidification technique in this study as depicted in Figure 1.

Evaluation of LRCH solid self-nanoemulsion

Powder Flow Properties

The powder of the two formulations shows excellent properties as depicted in Table 4.

**Table 4:** Flow properties of LRCH-SSNEDDS (SD from mean, n = 3) *: one-way ANOVA among triplicate experiments at a significance level of P < 0.05 LRCH-SSNEDDS: Lercanidipine hydrochloride - solid self-nano-emulsifying drug delivery system

Formula no.	The angle of repose θ	Compressibility index (Carr’s index)	Hausner’s ratio	Flow result
SSNEDDS1	29.09 ± 0.27	9.299 ± 0.102	1.086 ± 0.0015	Excellent
SSNEDDS2	26.80 ± 0.52	7.938 ± 0.113	1.034 ± 0.0020	Excellent

Drug Content

Table 5 depicts the results for the developed SSNEDDS formulation that is in acceptance with the official range of the British Pharmacopeia, as shown in the table (95% to 110%) [25].

**Table 5:** Drug content of SSNEDDS capsules formulations (SD, n = 3) *: one-way ANOVA among triplicate experiments at a significance level of P < 0.05 SSNEDDS: solid self-nano-emulsifying drug delivery system

Formula no.	Drug content %
SSNEDDS1	99.16 ± 0.531
SSNEDDS2	99.76 ± 0.125

**Figure 2:**
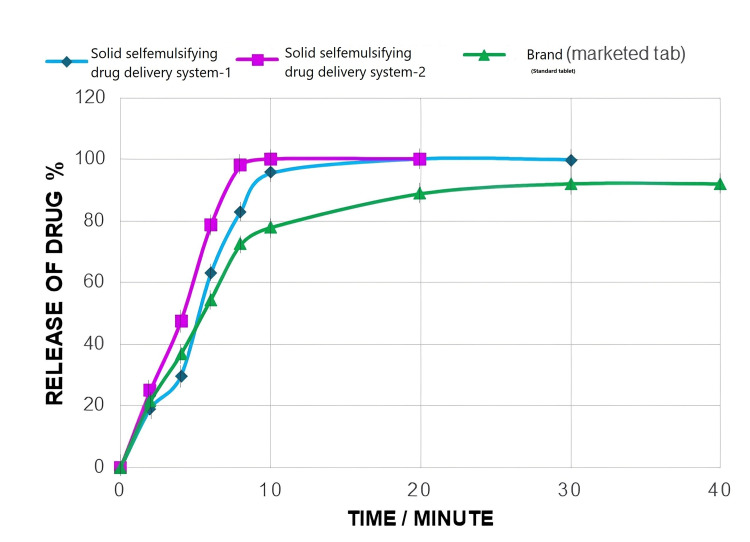
Dispersion rate profile of LRCH in SSNEDDS1 capsule, SSNEDDS2 capsule, and brand-marketed tablet The similarity f2 between the SSNEDDS2 capsule and the brand-marketed tablet is 40.65. LRCH: lercanidipine hydrochloride; SSNEDDS: solid self-nano-emulsifying drug delivery system

**Figure 3:**
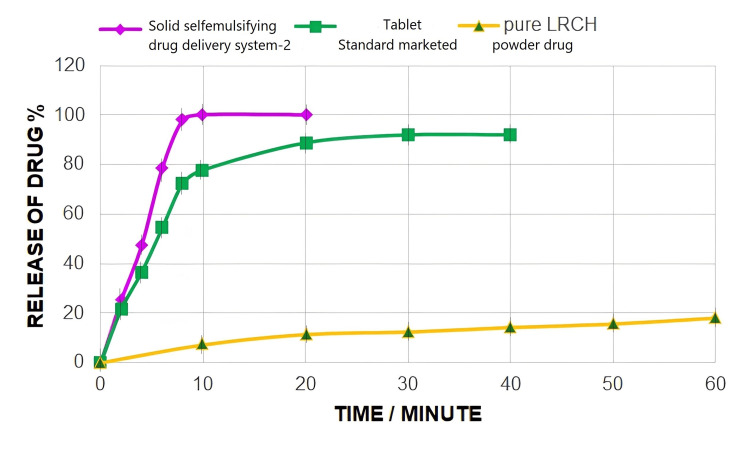
Dispersion rate profile of LRCH in SSNEDDS2 capsule, marketed tablet, and pure LRCH The similarity factor of the SSNEDDS2 capsule with pure LRCH is equal to 4.93. LRCH: lercanidipine hydrochloride; SSNEDDS: solid self-nano-emulsifying drug delivery system

The drug release study demonstrated how solid self-emulsifying nanoemulsion (SSNE) created nanodroplets when it was added to the medium. Although the lipophilic medication remains trapped in the oily droplets, the disintegration of the capsule shell releases the nanodroplet as a result, the drug release response resembles the way nanodroplets form. As depicted in Figures 2, 3, the dispersion (dissolution) rate is ranked in descending order as SSNEDDS2 > SSNEDDS1 > standard marketed tab > pure drug.

DSC

**Figure 4:**
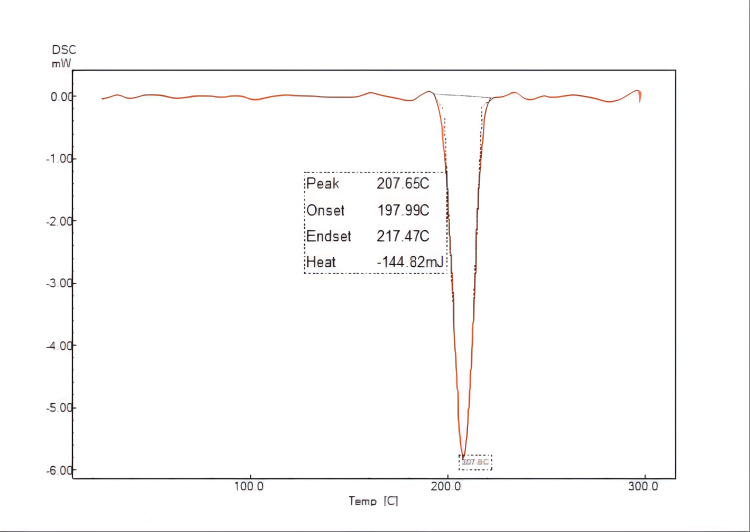
DSC analysis of pure LRCH DSC: differential scanning calorimetry; LRCH: lercanidipine hydrochloride

**Figure 5:**
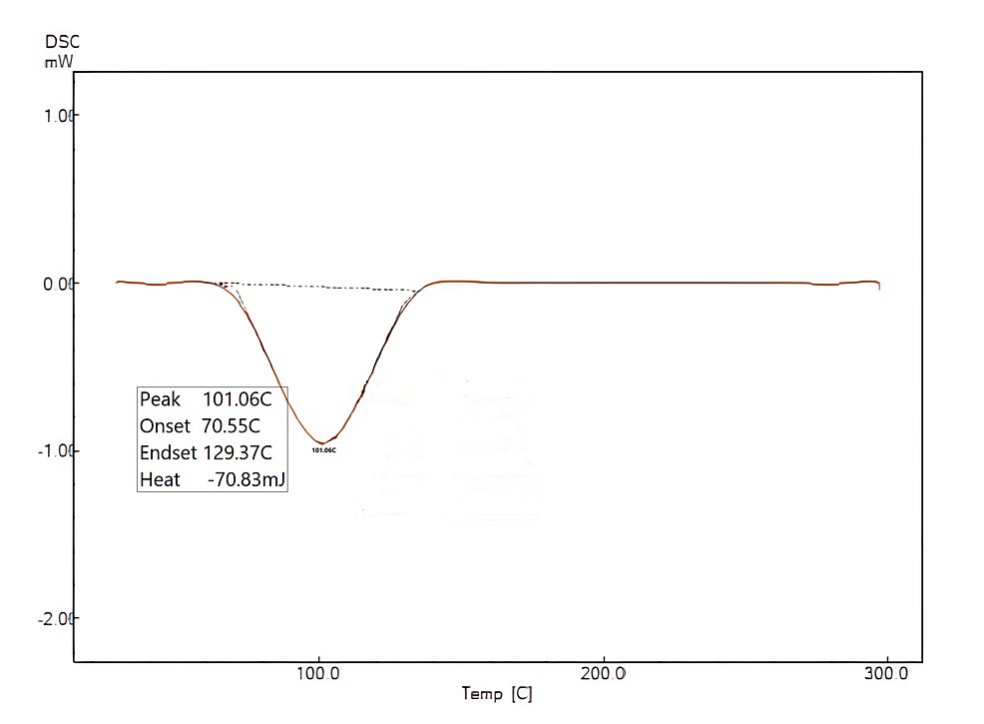
DSC analysis of the chosen LRCH-SSNEDDS formula DSC: differential scanning calorimetry; LRCH-SSNEDDS: lercanidipine hydrochloride - solid self-nano-emulsifying drug delivery system

The pure LRCH and the optimized LRCH-SSNEDDS were subjected to DSC analysis as depicted in Figures 4, 5. The DSC thermogram showed the pure drug's melting endotherm at 207.65°C which agrees with the melting point of the drug in reference. On the other hand, in the chosen formula, the endothermic peak disappeared indicating dispersion of the drug in oil results in reduced crystallinity [26].

Powder X-ray diffraction

**Figure 6:**
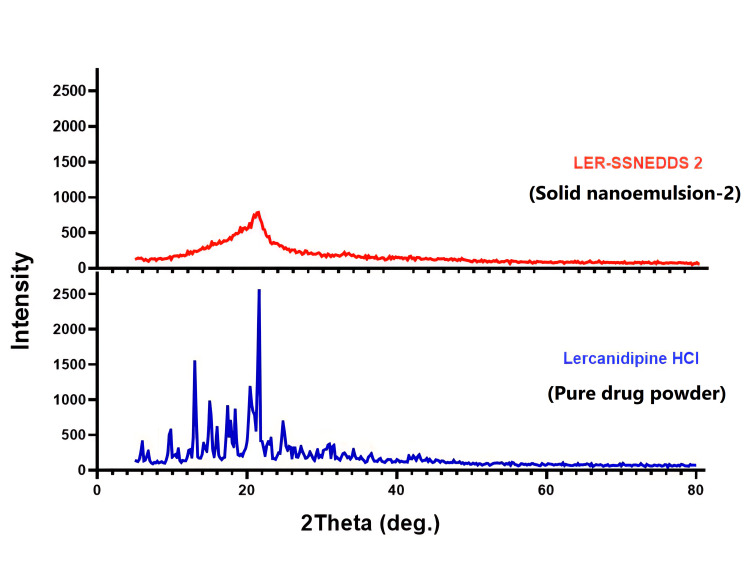
XRPD analysis of pure drug and chosen LRCH-SSNEDDS formula LRCH-SSNEDDS: lercanidipine hydrochloride - solid self-nano-emulsifying drug delivery system; XRPD: X-ray powder diffraction

The diffraction spectra of LRCH alone indicate that the medication is a highly crystalline powder with a sharp, powerful peak, as shown in Figure 6. On the other hand, the selected LRCH-SSNEDDS diffraction pattern is shown in this figure indicating a reduction in crystallinity.

## Discussion

Results showed that the two SSNEDDS formulations have excellent flow properties due to the characteristic flowability of the Avicel carrier and Aerosil 200. The drug content of SSNEDDS formulations was in acceptance with the official range of the British Pharmacopeia, as shown in Table 5 (95% to 110%) but SSNEDDS2 has a higher percentage of 99.761 ± 0.125 than 99.166 ± 0.531 of SSNEDDS1 indicated there is no LRCH precipitation or degradation.

As depicted in Figure 3, the dispersion rate is ordered as SSNEDDS2 > SSNEDDS1 > standard marketed tab > pure drug.

The faster dispersion was due to several reasons. First, the drug was dissolved in the SNEDDS system due to the solubilization effects of peppermint oil and Tween 20 which improved the hydrophilicity of LRCH. Second, the excellent nanoglobule size allowed for a larger surface area of nanoemulsion droplets. Third, a higher Smix ratio (Tween 20/PG) played a role in this faster release, especially when well-balanced with an oil ratio [27].

Also, there are several possibilities for further enhancement in the dispersion rate of LRCH from SSNEDDS formulations, besides reducing the particle size and increasing the surface area. The evidence presented suggests that the drug's amorphous nature, improved solubility, increased wettability, and rapid dispersibility occur in the dispersion medium with the assistance of various adsorbent mixtures. These mixtures have a large surface area, allowing for a faster dispersion of the drug into the aqueous phase [28].

Furthermore, the inclusion of additives such as aerosol 200 significantly improves the dispersion of LRCH. Many high-quality inorganic powders, such as Aerosil 200, have a particle size of approximately 15 nm in diameter. These powders also have a large surface area exposed, ranging from 100 m^2^/g to 400 m^2^/g.

Although the two formulations showed a complete dispersion of 100%, the SSNEDDS2 that contains Avicel 102 showed a faster dispersion of 100% within 10 minutes than SSNEDDS1 that contains Avicel 101 due to Avicel 102 generally exhibits a higher surface area and increased porosity in comparison to Avicel 101 because of its rough surface morphology and connected pore structure. A larger surface area facilitates higher drug adsorption and enhances interaction between the drug and the excipient, resulting in faster dispersion kinetics [29].

Figure 4 depicts a dispersion comparison of SSNEDDS2, standard tablet, and pure drug.

Despite different dosage form dispersion comparisons as the capsule shell needs time for disintegration, the results indicated that the drug dispersion rates of SSNEDDS2 capsules were faster than those of the marketed tablet (reaches 92% for 30 minutes and sustained after that) and pure drug (reaches 18.1% during 60 minute) due to the hydrophilic nature and excellent powder flow characteristics of Avicel improving particle wetting and quickening SSNEDDS component desorption, leading to spontaneous nanoemulsion production.

A comparison between two dissolution profiles was made using the similarity factor. As per the Food and Drug Administration (FDA) guidelines, the two profiles are deemed similar when the f2 value exceeds 50, ranging from 50 to 100. In this case, the resulting value of the similarity factor was less than 50, indicating a significant difference in the dispersion behavior [30,31]. Results show the value f2 <50.

The DSC thermogram showed the pure drug's melting endotherm at 207.65°C. On the other hand, in the chosen formula, the endothermic peak disappeared indicating dispersion of the drug in oil results in reduced crystallinity as depicted in Figures 4, 5.

The diffraction spectra of LRCH alone indicate that the medication is a highly crystalline powder with a sharp, powerful peak, as shown in Figure 6. On the other hand, the selected LRCH-SSNEDDS diffraction pattern is shown in this figure indicating a reduction in crystallinity.

Stability study results include monitoring of drug content and physical appearance for three months at three different temperatures (25°C, 40°C, and 50°C) indicating that there are no changes noticed in the drug content or appearance of both the LRCH-SNEDDS ampule and capsule. The results confirm that the developed SNEDDS formulation sustained stability for three months under the established conditions which confirms the fact that self-nanoemulsifying increases drug stability.

Limitations of the study

While the study successfully developed and evaluated a solid self-nanoemulsion formulation for LRCH, there are limitations that should be acknowledged, such as the sticky properties of the pure powder of the drug. In addition, although the solubility of the drug in oil is high, the miscibility with other constituents of the nanoemulsion determines the physical stability of the system and avoids phase separation.

## Conclusions

This study successfully prepared LRCH using the promising strategy of SSNEDDS as a hard gelatin capsule with a higher dispersion rate. Since it is lipid-based, there is an expected improvement in its bioavailability and stability compared to the brand-marketed tablet. The outcomes have the required and unique characteristics of the oral dose form, concluding the investigation. The LRCH-SSNEDDS oral hard gelatin capsule dosage form has great promise for the treatment of hypertension, particularly in patients with CKD.
